# Fibrolipomatous hamartoma of the sciatic nerve: an atypical case report

**DOI:** 10.3389/fradi.2025.1663742

**Published:** 2025-11-13

**Authors:** Yasser Hamdan Al Ghamdi, Saud Hussain Alawad, Fayka Karem, Mohammed J. Alsaadi

**Affiliations:** 1 Radiology and Medical Imaging Department, King Saud Medical City, Riyadh, Saudi Arabia; 2 Radiology and Medical Imaging Department, College of Applied Medical Sciences, Prince Sattam Bin Abdulaziz University, Al-Kharj, Saudi Arabia

**Keywords:** fibrolipomatous, hamartoma, nerve, rare, sciatic, MRI, surgery

## Abstract

Fibrolipomatous hamartoma is a rare benign overgrowth of tissue consisting of intermixed adipose and fibrous connective tissue within the epineurium. However, involvement of the sciatic nerve is exceptionally rare. We present the case of a 46-year-old female who exhibited a progressively enlarging mass in her right posterior thigh, accompanied by sciatica and gluteal pain. Clinical assessment and MRI revealed a large lesion along the sciatic nerve with characteristic features of fibrolipomatous hamartoma. MRI findings demonstrated characteristic features, including isointense (to fat) on T1-weighted images and hyperintense with fat suppression on short tau inversion recovery sequences, indicating a sciatic nerve fibrolipomatous hamartoma. The diagnosis was histopathologically confirmed following surgical excision. This case highlights the critical role of identifying specific MRI features of this rare entity to avoid unnecessary invasive interventional procedures. An accurate MRI-based diagnosis can significantly impact clinical decisions and improve patient care.

## 1 Introduction

Fibrolipomatous hamartoma (FLH), also referred to as nerve lipomatosis, perineural lipoma, intraneural lipoma, or lipofibromatous hamartoma, is a rare congenital malformation characterized by fibrofatty overgrowth within the epineurium of peripheral nerves (1–5). This abnormal proliferation typically produces fusiform swelling of the affected nerve and a pathognomonic “cable-like” appearance on imaging (2, 3). The median nerve and its branches are most frequently involved, accounting for more than 80% of reported cases. In contrast, sciatic nerve involvement is extremely rare and has been reported only in isolated cases (4–7). Such scarcity highlights the diagnostic challenge and clinical importance of documenting FLH in atypical anatomical locations. Historically, a range of overlapping terms—including intraneural lipoma, neural fibrolipoma, and lipofibromatous hamartoma—have been used to describe intraneural lipomatous lesions. However, the current World Health Organization (WHO) classification recognizes FLH as a distinct benign peripheral nerve tumour (3).

Recent molecular studies have raised the possibility that FLH belongs to the PIK3CA-related overgrowth spectrum (PROS). Somatic activating mutations in the PIK3CA gene, which result in hyperactivation of the PI3K/AKT/mTOR pathway, have been reported in cases of lipomatous overgrowth (8, 9). Although no universal genetic driver has been established, this potential molecular link may explain the congenital nature of FLH and its frequent association with macrodactyly. Clinically, FLH can present at any age, from infancy to the seventh decade of life. Still, it is most often diagnosed in adolescence or early adulthood and appears more commonly in females (2). While its pathogenesis is primarily congenital, additional factors such as trauma or chronic nerve irritation may accelerate lesion growth (3–5).

From a diagnostic perspective, ultrasound (US) and computed tomography (CT) may provide supportive information, but magnetic resonance imaging (MRI) remains the gold standard owing to its superior soft-tissue contrast and ability to characterize nerve architecture (4). The characteristic MRI appearance—described as “coaxial cable-like” on axial views and “spaghetti-like” on longitudinal views—allows a confident, non-invasive diagnosis in most cases, often obviating the need for biopsy (5, 6). In this case report, we present a rare FLH of the sciatic nerve and emphasize the importance of recognizing its MRI features. Prompt and accurate imaging-based diagnosis can prevent unnecessary invasive procedures and guide appropriate management strategies.

## 2 Case presentation

### 2.1 Clinical findings

A 46-year-old female patient who was experiencing sciatica and buttock pain underwent evaluation at our hospital for a palpable mass in the region of the right upper posterior thigh. The swelling had gradually increased significantly over the past few years. Intermittent nonradiating pain was worse in the sitting position. The patient also had oedema in the right gluteal region. On examination, the patient was alert and hemodynamically stable. She was obese but had no leg oedema or lymphadenopathy. Examination revealed a nontender, circumscribed swelling measuring 2.9 × 2.6 cm with no skin changes observed. Neurological examination revealed normal motor power (5/5) in both lower limbs, preserved muscle tone, and intact deep tendon reflexes. Sensory assessment showed no dermatomal loss. Straight-leg raise was negative. However, intermittent local tenderness and discomfort were elicited over the posterior thigh mass.

### 2.2 Diagnostic assessment

X-ray imaging of the lumbosacral and gluteal regions showed no significant findings. A subsequent high-resolution MRI scan revealed a mass measuring 2.9 × 2.6 cm along the path of the right sciatic nerve. Magnetic resonance imaging (MRI) was performed utilising a GE Signa 1.5T MRI scanner (GE Healthcare, USA) equipped with a dedicated body coil. Axial and coronal images were acquired with a slice thickness of 3 mm, an interslice gap of 3 mm, and a field of view (FOV) measuring 340 mm. The imaging protocol included T1-weighted spin-echo, T2-weighted fast spin-echo, and Short Tau Inversion Recovery (STIR) sequences in both planes. Diffusion-weighted imaging (DWI) and Apparent Diffusion Coefficient (ADC) maps were generated using b-values of 0 and 800 s/mm^2^. The lesion was measured in both planes. The lesion appeared isointense to fat on both T1-weighted axial and coronal images (Supplementary Figure S1). Short tau inversion recovery (STIR) images in axial and coronal planes (Supplementary Figure S2) demonstrated a large mass with hyperintense signals and a fibrillar appearance, with fat signals within the area being suppressed. Contrast-enhanced sequences were not obtained due to patient refusal; however, non-contrast MRI demonstrated pathognomonic features, allowing a confident diagnosis of fibrolipomatous hamartoma. The MRI features were consistent with sciatic nerve FLH. Histopathological analysis confirmed fibrolipomatous hamartoma, demonstrating mature adipocytes and fibrous connective tissue infiltrating the epineurium without atypia. Due to technical limitations, representative photomicrographs are not available for publication.

### 2.3 Therapeutic intervention

Surgical excision of the tumour was recommended as treatment, but the patient refused to undergo surgery and was subsequently advised to have regular follow-ups. She returned to the outpatient clinic with worsening right gluteal pain one year after her initial presentation. A surgical excision was then performed, and histopathological analysis confirmed that the mass was an FLH. The patient was followed up clinically at regular intervals for 12 months after surgical excision. No postoperative MRI was performed. At the last follow-up, one year after surgery, the patient remained completely asymptomatic, with no recurrence of pain or palpable mass and full return to daily activities. A summary timeline of the clinical course is presented in Supplementary Figure S3.

## 3 Discussion

Fibrolipomatous hamartoma (FLH) is a rare congenital malformation of peripheral nerves characterised by excessive growth of adipose and fibrous tissue within the epineurium. More than 80% of cases involve the median nerve, whereas involvement of the sciatic nerve is extremely rare and has been documented only in isolated case reports (1–5). This rarity emphasises the diagnostic challenge and clinical significance of reporting unusual presentations.

The aetiology of FLH remains incompletely understood. The leading theory proposes it as a developmental abnormality of the nerve sheath with abnormal fibrofatty infiltration (4–6). Trauma and ongoing nerve irritation are thought to be secondary factors that accelerate lesion growth rather than primary causes (7, 8). Recent molecular research has identified somatic activating mutations in the PIK3CA gene in some cases of FLH, linking dysregulation of the PI3K/AKT/mTOR pathway to FLH and categorising it within the PIK3CA-related overgrowth spectrum (PROS) (8–10). However, findings are inconsistent, and not all lesions display identifiable mutations, highlighting genetic variability. Further comprehensive molecular studies are needed to better understand the mechanisms underlying FLH.

Histologically, FLH is characterised by mature adipocytes and fibrous stroma infiltrating the epineurium while preserving nerve fascicles. This distinguishes it from intraneural lipomas, which are encapsulated and displace rather than infiltrate fascicles. The extent of fatty infiltration varies and may explain the heterogeneity of imaging appearances and clinical manifestations. Although benign, lesion enlargement can cause compressive neuropathy and functional impairment, as observed in our patient.

FLH typically occurs in adolescence or early adulthood and shows a slight female predominance (7–11). However, epidemiological studies indicate no clear racial or geographic preference, and some series have reported an almost equal sex distribution (12). Clinically, patients often present with a slow-growing, painless mass. Over time, compressive symptoms such as neuropathic pain, motor weakness, or sensory disturbances may develop. In the sciatic nerve, symptoms can resemble common conditions like lumbar disc herniation or sciatica, leading to diagnostic delays. In our case, progressive swelling and sitting-agonised sciatic pain reflected the natural history of FLH seen in other nerve distributions. MRI remains the primary diagnostic tool for FLH due to its superior soft tissue contrast and ability to delineate nerve structures (5, 6, 11, 13). The typical features include the “coaxial cable-like” pattern on axial images and the “spaghetti-like” appearance on longitudinal images, which reflect the presence of interspersed fibrous and fatty tissues within hypertrophic fascicles. On T1-weighted sequences, FLH shows hyperintense fatty signals interspersed with hypointense fibrous strands, while T2-weighted and fat-suppressed sequences highlight fibrous components and oedema. Contrast-enhanced MRI can be helpful, although it is not always performed; minimal or no enhancement after gadolinium helps to differentiate FLH from malignant peripheral nerve sheath tumours or inflammatory neuropathies (2, 5, 9, 12). Advanced MRI sequences, including DWI, proton density, and gradient echo, assist in better characterisation of fibrofatty infiltration. The absence of significant gadolinium enhancement is a key feature that distinguishes FLH from malignant peripheral nerve sheath tumours (5–7, 13). Other modalities, such as ultrasound (US), can complement an MRI diagnosis of FLH. US is an accessible alternative diagnostic method that provides a dynamic image of FLH, which appears as a hyperechoic lesion due to its fatty tissue component. The US image may show diffuse or focal nerve enlargement with distorted fascicular architecture. In Colour Doppler mode, FLH may exhibit reduced or normal vascularity, distinguishing it from tumours with increased vascularity. The US image can also provide a real-time assessment of nerve mobility and any compression effects (7, 8). Diagnostic imaging can also guide surgical biopsy by identifying the precise location and extent of the lesion (5–8). Nevertheless, although imaging is crucial for diagnosis, histopathological examination remains the definitive diagnostic modality for FLH, as it confirms the infiltration of fibrous and fatty tissue and preserves nerve bundles (7, 13, 14).

Once FLH is diagnosed, management depends on symptom severity, nerve involvement, and patient preference. Different options are considered, including both surgical and nonsurgical methods. For asymptomatic or mildly symptomatic patients, nonsurgical treatment is advised; this usually involves pain relief with nonsteroidal anti-inflammatory drugs (NSAIDs) or corticosteroids. Physiotherapy is also recommended to relieve pressure symptoms and preserve nerve function (6). Surgery is indicated for more severe compressive neuropathy or worsening symptoms. One surgical option is decompression surgery, which can relieve symptoms without damaging nerve function. Nerve grafting is also considered in cases with extensive nerve involvement (5–7, 14).

Recognising the imaging features of FLH is crucial, especially at unusual sites such as the sciatic nerve, where its rarity may lead to misclassification as soft-tissue sarcoma, liposarcoma, or nerve sheath tumour. Accurate diagnosis prevents unnecessary biopsy or radical resection and helps to preserve nerve function. Although FLH is benign and non-malignant, its progressive growth can severely impact quality of life through compressive neuropathy. Greater awareness among clinicians and radiologists is therefore vital to ensure prompt and accurate diagnosis (11–14).

In our case, the patient initially refused surgery, underscoring the challenges of patient compliance in managing benign conditions. However, surgical excision was performed after worsening symptoms, and histopathology confirmed the diagnosis of FLH. Postoperative outcomes were favourable, with the patient reporting a complete resolution of symptoms and resumption of regular activities.

This case illustrates some of the most important clinical and radiological aspects of FLH:

1. Rare nerve involvement: Sciatic nerve lipomatosis is exceptionally rare, making this case valuable to the literature.
2. MRI remains the diagnostic cornerstone, obviating the need for invasive biopsy and guiding treatment decisions.
3. Surgical excision is effective for symptom relief and facilitates functional restoration, particularly in cases of significant symptom progression.

In conclusion, this case report shows that sciatic nerve FLH, a rare condition, can cause sciatica and should be a key consideration in differential diagnosis. Advanced MRI techniques can distinguish this condition from other causes of an enlarged sciatic nerve, allowing diagnosis without a biopsy. Awareness of this rare entity can prevent patients from undergoing unnecessary biopsies or surgical interventions, thus significantly improving patient care.

## Data Availability

The original contributions presented in the study are included in the article/Supplementary Material, further inquiries can be directed to the corresponding author.
