# Supplementary material for: Fibrolipomatous hamartoma of the sciatic nerve: an atypical case report
Source: Front Radiol. 2025 Nov 13;5:1663742. doi: 10.3389/fradi.2025.1663742 (PMC12657156; doi:10.3389/fradi.2025.1663742)
Supplement: Supplementary file 1 [file Datasheet1.docx]

Supplementary Material

# Supplementary Figures

**Supplementary Figure 1:** A, Axial T1 image eliciting oval-shaped isodense lesion between the right ischial tuberosity and the right gluteus maximus muscle (white arrow). B, Coronal T1 images show a fusiform iso-to-hypertense lesion noted at the course of the right sciatic nerve (white arrow)

**Supplementary Figure 2:** A, Axial STIR images show suppression of the fat signals within the tumour (white arrow). B, the coronal STIR image shows a hyperintense lesion with a fibrillar appearance and the suppression of fat signals within the tumour (white arrow).

**Supplementary Figure 3:** The timeline summarises the patient’s initial presentation, physical examination, diagnosis imaging, management discussion, treatment plan and follow-up.


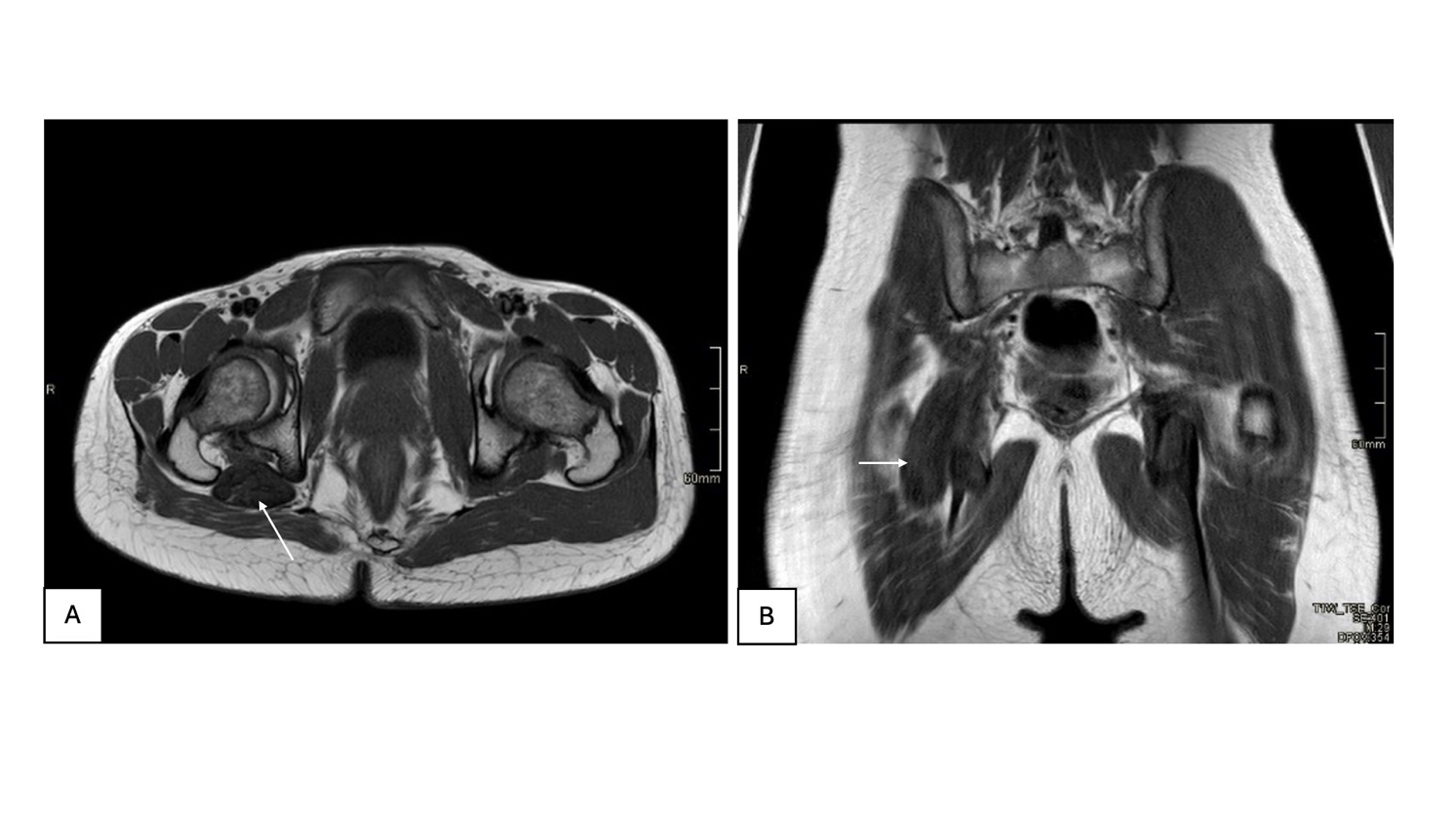


Figure 1.


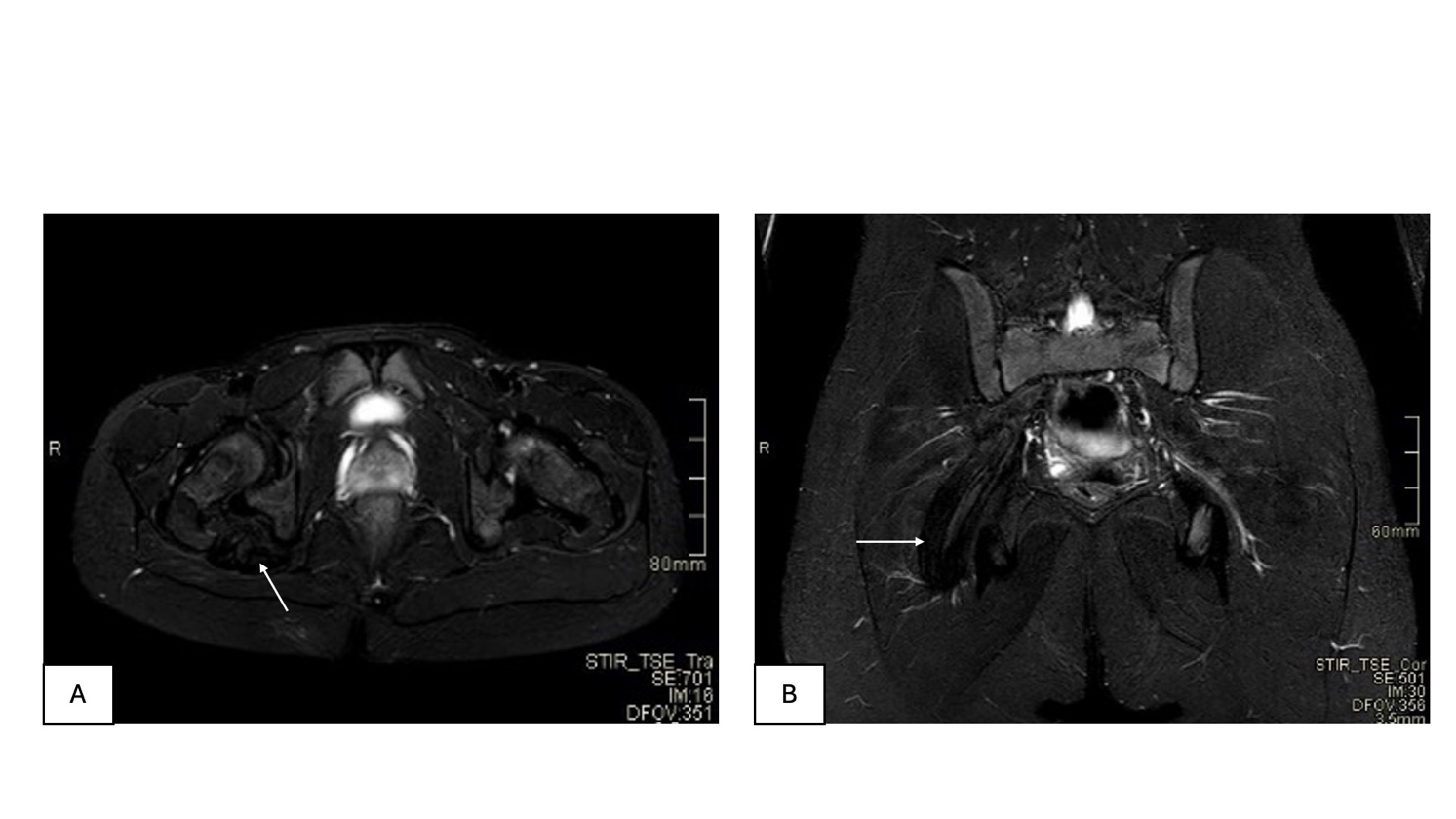


Figure 2.


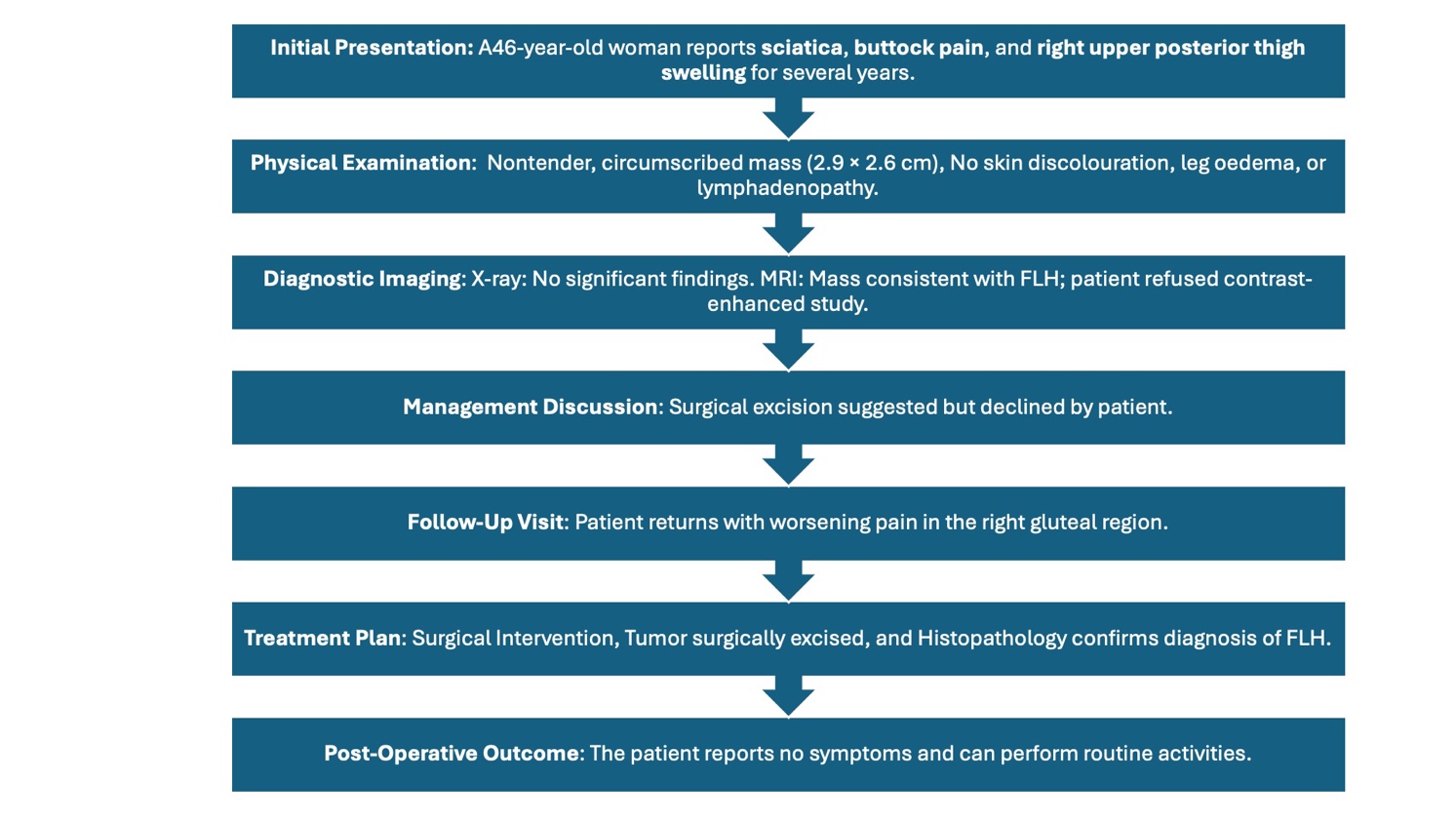


Figure 3.
